# Living Porous Ceramics for Bacteria‐Regulated Gas Sensing and Carbon Capture

**DOI:** 10.1002/adma.202412555

**Published:** 2024-12-10

**Authors:** Alessandro Dutto, Anton Kan, Zoubeir Saraw, Aline Maillard, Daniel Zindel, André R. Studart

**Affiliations:** ^1^ Complex Materials Department of Materials ETH Zürich Zürich 8093 Switzerland; ^2^ Laboratory of Physical Chemistry ETH Zürich Zürich 8093 Switzerland

**Keywords:** bio‐sensing, engineered living materials, microorganisms, scaffolds, synthetic biology

## Abstract

Microorganisms hosted in abiotic structures have led to engineered living materials that can grow, sense, and adapt in ways that mimic biological systems. Although porous structures should favor colonization by microorganisms, they have not yet been exploited as abiotic scaffolds for the development of living materials. Here, porous ceramics are reported that are colonized by bacteria to form an engineered living material with self‐regulated and genetically programmable carbon capture and gas‐sensing functionalities. The carbon capture capability is achieved using wild‐type photosynthetic cyanobacteria, whereas the gas‐sensing function is generated utilizing genetically engineered *E. coli*. Hierarchical porous clay is used as a ceramic scaffold and evaluated in terms of bacterial growth, water uptake, and mechanical properties. Using state‐of‐the‐art chemical analysis techniques, the ability of the living porous ceramics are demonstrated to capture CO_2_ directly from the air and to metabolically turn minute amounts of toxic gas into a benign scent detectable by humans.

## Graphical abstract

Porous ceramics colonized by bacteria can achieve self‐regulated, programmable carbon capture and gas‐sensing functions. The porosity of the ceramic scaffold enables autonomous nutrient transport through capillary forces, enabling microbial growth without external energy. Using wild‐type cyanobacteria and engineered *E. coli*, living porous ceramics are created to capture CO_2_ directly from the air and convert toxic gases into human‐detectable benign scents.

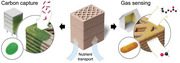

## 1 Introduction

Engineered living materials (ELMs) harness the biological activity of microorganisms to imbue synthetic structures with adaptive, sensing, and decision‐making functionalities.^[^ ^1^ ^]^ Examples of ELMs include self‐healing concrete,^[^ ^2^ ^]^ living sensors for water quality control,^[^ ^3^ ^]^ regenerative robotic skins,^[^ ^4^ ^]^ and self‐lubricating contact lenses.^[^ ^5^ ^]^ The microorganisms responsible for these living properties vary from bacteria to algae and fungi, which can be harvested directly from the environment or genetically engineered for the function of interest. Engineered microorganisms can harness biological activity in a programmable manner to add diverse functionalities to ELMs, leading to applications in catalysis, energy conversion, sensing, electronics, and biomedicine.^[^ ^1^, ^6^ ^]^ Despite these remarkable examples, the potential of ELMs to generate and keep living functionalities strongly depends on the host material. For the ELM to be functional and remain alive it is essential to provide a host material for the cells to proliferate and maintain their metabolic activity. While hydrogels and particle networks have been widely used as host structures, they may limit the growth of microorganisms and their access to oxygen and nutrient‐containing media. This calls for novel scaffold materials that promote colonization by microorganisms, supply nutrients and harness the resulting biological activity of the engineered living material.

Porous ceramics have been extensively used as scaffolds for the in‐growth of cells for tissue engineering applications.^[^ ^7^ ^]^ By tuning the porosity and pore size of the structure, it is possible to create scaffolds that promote cell proliferation while ensuring sufficient nutrient supply for their growth and biological activity. This is often achieved using scaffolds with a hierarchical porous architecture, in which small pores below 10 µm generate the high surface area required for cell adhesion^[^ ^8^ ^]^ whereas large pores with 50–1000 µm provide the high permeability needed for vascularization and continuous supply of oxygen and nutrients.^[^ ^9^ ^]^ A broad range of techniques has been utilized for the fabrication of hierarchical porous ceramics, including 3D printing, foaming, emulsion templating, and freeze casting.^[^ ^10^ ^]^ 3D printing of wet foams and emulsions is a particularly suitable approach to independently control pore sizes at multiple length scales.^[^ ^11^ ^]^ Using these hierarchical porous ceramics as host scaffolds for wild‐type or engineered microorganisms should lead to mechanically robust living materials with high metabolic activity and tunable functionalities.

The functionalities of engineered living materials are primarily controlled by the activity of the microorganisms hosted within an abiotic structure. Depending on the function of interest, the desired activity might already be encoded in wild‐type microorganisms or may require genetic modification to create new microbial strains. Carbon capture is an example of a functionality of ELMs that has been achieved using wild‐type photosynthetic microorganisms, such as microalgae and cyanobacteria.^[^ ^12^ ^]^ In this case, the natural metabolism of the microorganism is harnessed to convert CO_2_ from the air into organic molecules that can be stored within the cells. Beyond native species, the range of functionalities of ELMs can be significantly extended by programming the hosted microorganisms with synthetic biology tools.^[^ ^13^ ^]^ Illustrative examples are living sensors that contain bacteria engineered to detect, transduce, and amplify chemical signals using programmable genetic circuits.^[^ ^6^, ^14^ ^]^ Overall, the wide spectrum of microorganisms, synthetic biology tools, and manufacturing processes currently available provide extensive opportunities to develop living materials engineered with functions unavailable to traditional materials.

Here, we develop and study living porous ceramics with gas‐sensing and carbon‐capture functionalities regulated by metabolically active bacteria. Inspired by the water transport mechanism of trees, the porous structure is designed to pump and distribute nutrient‐rich water for the growth and metabolism of microorganisms embedded within the living material. First, we study the water transport, bacterial proliferation, and mechanical stability of the porous ceramic structure. Next, ureolytic bacteria are used to mechanically reinforce the porous structure via a room‐temperature biocementation process. The porous ceramics are then loaded with photosynthetic microorganisms and investigated in terms of carbon capture capabilities. Finally, engineered bacteria are implemented in the porous ceramics to create a living material that indicates the presence of a toxic gas by generating a benign smell detectable by the human nose.

## 2 Results and Discussion

**Figure 1:**
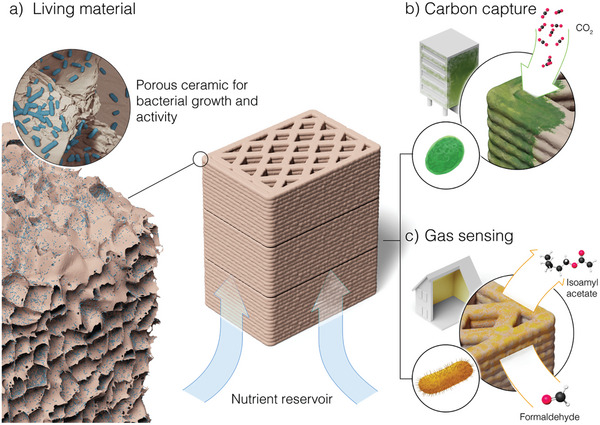
Living porous ceramic for carbon capture and gas sensing. The potential use of living ceramics as building materials is highlighted. a) The porous ceramic serves as a scaffold for the growth and activity of specific microorganisms. b) Carbon capture and c) gas‐sensing capabilities are achieved by using wild‐type photosynthetic cyanobacteria or engineered microorganisms designed as biosensors.

The envisioned living porous ceramics should display a large fraction of open, interconnected pores to enable the growth of the microorganisms and continuous intake of nutrient‐rich culture medium (**Figure** **1**). Bacterial growth is facilitated by selecting ceramic particles featuring biocompatible and hydrophilic surface chemistry.^[^ ^15^ ^]^ To fulfill this criterion, naturally occurring clay was chosen to be the solid phase of the porous ceramic. Besides its environmentally friendly nature, clays are widely available and have been extensively used in the building industry. Moreover, several processing techniques exist for the processing of clay‐based ceramics with controlled porosity and pore sizes (Figure 1a).

Ceramics with carbon capture and gas‐sensing functionalities can be created by growing bacteria with the desired metabolic features within the porous structure (Figures 1b,c). This is possible using either wild‐type or genetically modified microorganisms. As an essential natural process of the Earth's carbon cycle, the consumption of CO_2_ during photosynthesis is a metabolic feature found in a broad range of autotropic wild‐type microorganisms. For gas sensing, the biological activity of the microorganisms needs to be engineered for the detection, transduction, and amplification of the molecules of interest, which so far has mostly been demonstrated for chemicals dissolved in liquids.^[^ ^6^, ^16^ ^]^

**Figure 2:**
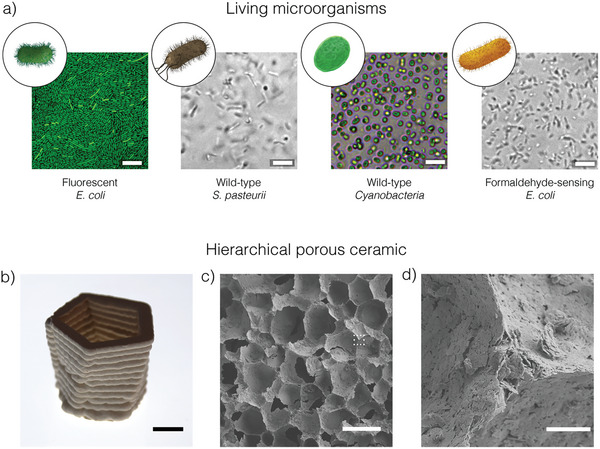
Microorganisms and printed structures are used for the creation of living porous ceramics. a) Illustrations and optical microscopy images of *E. coli* engineered to produce green fluorescent protein (GFP), the wild‐type *Sporosarcina pasteurii*, the wild‐type cyanobacteria *Synechococcus sp*., and *E. coli* engineered to sense the hazardous gas formaldehyde. b) 3D printed porous ceramic displaying (c) macropores and d) micropores at different length scales. Scale bars: 10 µm in (a), 1 cm in (b), 100 µm in (c), and 5 µm in (d).

To create and study the proposed living porous ceramics, four microorganisms were selected: i) an *Escherichia coli* strain engineered to produce green fluorescent protein (GFP), ii) a wild‐type ureolytic soil bacterium *Sporosarcina pasteurii*, iii) a wild‐type, cyanobacteria *Synechococcus sp*. (PCC 7002), and iv) an *Escherichia coli* strain engineered for gas sensing (**Figure** **2**a). The GFP‐producing *E. coli* was used as a model microorganism to investigate the growth and colonization of the porous structure by bacteria. Wild‐type *S. pasteurii* and *Synechococcus sp*. are two broadly available microorganisms that were used for mechanical reinforcement via biocementation and for carbon capture through photosynthesis, respectively. The gas‐sensing *E. coli* strain was engineered to detect low concentrations of toxic gas and translate this chemical signal into benign molecules identifiable by humans.

Living engineered materials were created by hosting the selected microorganisms in clay‐based ceramics with open hierarchical porosity. Such clay‐based porous ceramics were prepared from air bubble templates using a foaming technique previously reported in the literature.^[^ ^17^ ^]^ In this technique, a wet foam is first created by incorporating air bubbles in a suspension of surface‐hydrophobized clay particles. Several approaches can be used for the surface hydrophobization of initially hydrophilic oxide particles.^[^ ^18^ ^]^ The adsorption of the hydrophobized clay particles onto the surface of the air bubbles allows for direct drying of the wet foam, which is then sintered at 1000 or 1150°C for 2h to generate a ceramic scaffold with porosity of 80–90% (see Experimental Section).

Porous ceramics obtained via this foaming route display a hierarchical family of pores with sizes in the ranges of 20–130 µm and 20–80 nm.^[^ ^17b^ ^]^ The macropores originate from the templating air bubbles, whereas the micropores arise from the interstices between the clay particles. By increasing the sintering temperature from 1000 to 1150°C, it is possible to reduce the fraction of 20–80 nm micropores present in the macropore walls while keeping the overall porosity of the structure at 90% (Figure S1, Supporting Information). Besides lowering the microporosity, sintering also reduced the average size of macropores from ≈60 to 40 µm, which might be attributed to the densification and shrinkage of the macropore walls. Such porous structures have been shown to absorb water through capillary forces due to the favorable wetting of the clay surface. This leads to a wicking effect that can be exploited for thermal insulation or cooling of architectural elements.^[^ ^17b^ ^]^ Because the wet foams can also be 3D printed, this processing technique allows for the manufacturing of complex‐shaped porous structures with three‐level hierarchical porosity that are not accessible using conventional fabrication techniques (Figures 2b–d).

To provide a suitable home for microorganisms, porous ceramics need to fulfill three specific requirements. First, the porous structure should provide the nutrients and aqueous medium necessary for cellular growth and metabolic activity. Second, the selected bacteria must grow, colonize, and remain metabolically active in the water‐filled porous structure. Finally, the bacteria‐laden porous ceramic needs to be sufficiently strong to withstand the mechanical loading expected in the intended application. The suitability of the foam‐derived structures to meet these criteria was evaluated by conducting experiments that assessed the hierarchical porous clay in terms of liquid uptake, bacterial growth, and mechanical stability.

**Figure 3:**
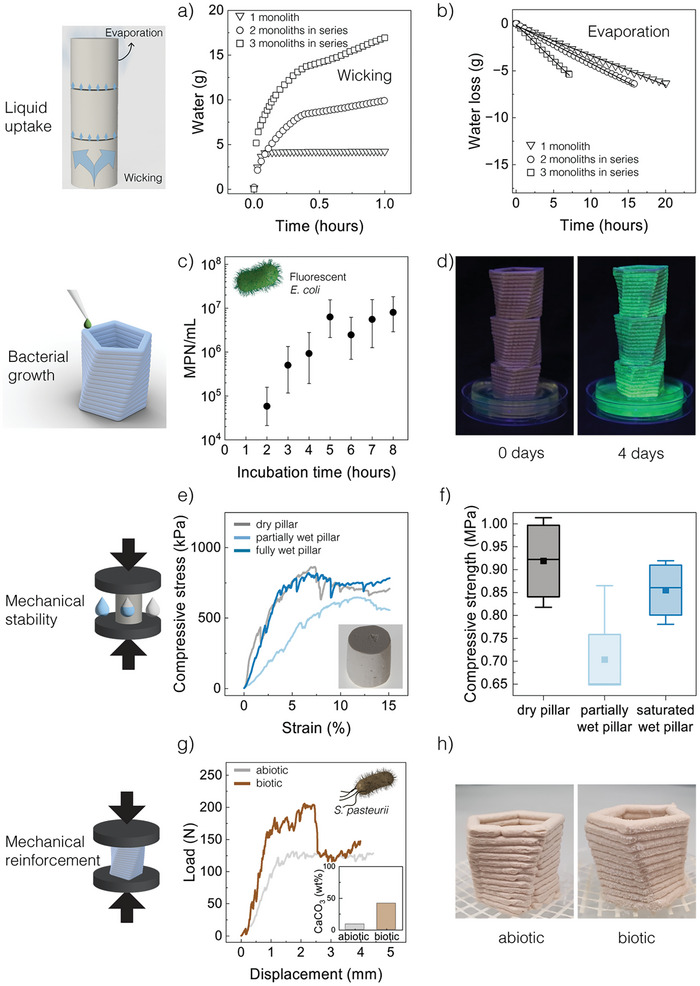
Porous ceramics as scaffolds for metabolically active bacteria. a) Wicking and b) evaporation behavior of the porous monoliths quantified by, respectively, the uptake and loss of water as a function of time. c) The density of fluorescent *E. coli* in the porous ceramics as a function of incubation time. d) Photographs of stacked 3D printed monoliths under UV light, illustrating the homogeneous distribution of fluorescent bacteria (green) achieved after 4 days of incubation. e,f) Compression tests were performed on cylindrical scaffolds in the dry, partially wet, and fully wet states. Plots e,f) display representative stress‐strain curves and box‐plots of the compressive strength obtained for the tested samples. g,h) Mechanical reinforcement of pre‐sintered ceramic scaffolds by bacterial‐induced biocementation. g) Representative stress‐strain response and carbonate fraction (inset) of biocemented scaffolds compared to a control sample without bacteria. h) Photographs of the abiotic and biotic monoliths after biocementation for 3 days.

The ability of the porous structure to take‐up liquid and to make this nutrient‐rich medium available for bacterial growth depends on the wicking kinetics and the evaporation rate of the liquid inside the pores. The liquid medium will remain in the pores if the wicking rate throughout the structure is equal to or higher than the evaporation rate across the surface of the monolith. To evaluate whether the porous ceramic satisfies this requirement, we measured the wicking and evaporation rates of the aqueous medium through representative casted monoliths (**Figures** **3**a,b). The measurements were performed on single and stacked monolithic units in contact with a water reservoir to mimic the possible arrangement of porous bricks in the envisioned living building material (Figure 1).

The experiments revealed that the transport of liquid through the tested monoliths is up to two orders of magnitude faster than the evaporation of the liquid medium across the sample surface (Figures 3a,b). For a single‐unit monolith, wicking occurs predominantly within the first 5 min at an average rate of 47 mL h^−1^ and follows the square‐root time dependence expected from the Washburn model for wetting liquids.^[^ ^17b^ ^]^ By contrast, evaporation takes place at a constant rate in the range of 0.32–0.76 mL h^−1^ over 18 h, which is typically observed when the process is controlled by a liquid film formed on the surface of the monolith.^[^ ^19^ ^]^ Normalization of the evaporation rate with respect to the exposed surface area leads to comparable values of 117, 97, and 125 mL h^−1^ m^−2^ for the single‐, 2‐unit, and 3‐unit monoliths, respectively (Table S1, Supporting Information). These comparable rates confirm that the removal of water from the monolith is determined by its exposed surface area, which can be tuned by the geometry of the complex‐shaped structure. Overall, our analysis of the experimental data provides useful guidelines for the design of porous monoliths that can remain filled with nutrient‐rich liquid, while allowing for constant liquid circulation driven by wicking (Supporting Information). Notably, wicking also happens across stacked monoliths, indicating that the liquid can effectively bridge between vertically adjacent units. The experiments show that the wicking rates for the initial water uptake decrease with the number of stacked monoliths, which suggests that mass transport across the liquid bridge becomes the rate‐limiting step in such multi‐unit systems.

Interestingly, when ≈40% of the water capacity of the stacked monolith system is reached, its weight continues to increase at a rate proportional to the number of additional monoliths (Figure S2, Supporting Information). This phenomenon might be related to the lateral growth of the liquid bridge formed between monoliths, which enables continuous water uptake in 2‐unit and 3‐unit stacked monoliths beyond the initial imbibition phase. Such an interpretation is supported by the fact that the single monolith (no liquid bridge) does not show further water uptake after the initial wicking. The lower rate of wicking observed for 2 and 3 stacks at this later stage might arise from the fact that the menisci of the liquid bridge formed between monoliths display a larger radius of curvature and therefore should grow at a lower rate compared to the menisci formed inside the porous monoliths.

The favorable wicking conditions enabled by the porous structure open the possibility of infiltrating and colonizing the ceramic scaffold with microorganisms. To demonstrate this, we designed an experiment in which a culture medium loaded with GFP‐producing bacteria is infiltrated into a stack of piled monoliths through the action of capillary forces (Figures 3c,d). The bacterial colonization of the scaffolds through this mechanism was quantified by measuring the total number of cells in the material over time using the most probable number method.^[^ ^20^ ^]^ The results showed that the density of bacteria in the scaffold grew exponentially up to 10^6^–10^7^ mL^−1^ of liquid within the first 5 h of the experiment. This suggests that the solid phase of the scaffold does not prevent the bacteria from proliferating as they would in a nutrient‐rich liquid environment. Such proliferation behavior allows for extensive colonization of stacks of porous monoliths, as visually demonstrated by an experiment in which GFP‐producing *E. coli* were used to infiltrate scaffolds through wicking and bacterial growth (Figure 3d).

While the high porosity of the scaffolds is crucial to enable bacterial colonization, it is also important that the porous structure is sufficiently strong to withstand the mechanical stresses expected during infiltration and final application. To evaluate this, we performed compression tests on porous cylindrical samples in three distinct representative conditions: fully dry, partially wet, and fully wet (Figures 3e,f). With an average compressive strength of 0.9 MPa and an average elastic modulus of 16 MPa (Figure S3, Supporting Information), the fully dry scaffolds reached mechanical properties comparable to standard dense bricks.^[^ ^21^ ^]^ The partially wet scaffold showed the lowest mechanical strength (0.7 MPa), which may result from the capillary forces that develop within the structure when water menisci are formed between the particles. This implies that the wicking process is a critical phenomenon for the mechanical integrity of the porous structure.

To ensure that the porous structure is not damaged by wicking‐induced capillary forces, the ceramic scaffold can be further reinforced through a bacteria‐driven biocementation process at ambient temperature. We demonstrate this reinforcing effect using wild‐type *Sporosarcina pasteurii*, which are soil bacteria that can induce the precipitation of calcium carbonate in the structure if supplied with nutrients, urea, and calcium ions from a mineralizing aqueous solution (Figures 3g,h). These bacteria produce the enzyme urease, which hydrolyses urea and thereby generates CO_3_^2−^ and OH^−^ ions in the solution.^[^ ^22^ ^]^ The pH increase resulting from this reaction leads to the oversaturation of the solution, thus inducing precipitation of calcium carbonate particles. By immersing a pre‐sintered, 3D‐printed porous ceramic for 3 days in a solution of urea and calcium, it is possible to increase the stiffness and the load‐bearing capacity of the printed structure by, respectively, 55 and 78%, compared to an abiotic control sample (Figure 3g). Such a strengthening effect correlates with the calcium carbonate content of the scaffold, which is, respectively, 9 and 42% in the abiotic and biotic samples after the biocementation process (Figure 3g, inset).

**Figure 4:**
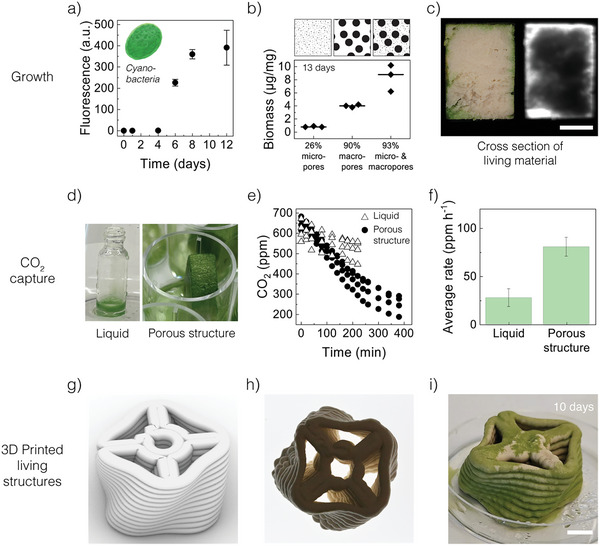
Living porous ceramics with carbon‐capturing cyanobacteria. a) Growth of cyanobacteria in the hierarchical porous ceramic quantified by the chlorophyll autofluorescence as a function of time. b) Effect of the porous architecture on the biomass generated through bacterial photosynthesis. c) Photograph (left) and fluorescence image (right) of the cross‐section of a hierarchical porous ceramic after 8 days of bacteria growth. d) Samples were used to quantify the CO_2_ capture ability of the bacteria‐laden hierarchical porous monolith relative to a liquid culture. e) Evolution of the CO_2_ concentration in the gas phase during photosynthesis in porous and control samples. The data show three replicas for each experimental condition (liquid and porous structure). f) The average rate of CO_2_ capture was measured for the hierarchical porous structure compared to the reference sample. The averages and standard deviations displayed in this plot were calculated from the three replicas shown in (e). Normalization with the envelope surface area gives 0.29 and 0.17 µmol m^−2^ s^−1^ for the porous structure and the reference sample, respectively. g–i) Rendering (g) and realization of a 3D printed hierarchical porous structure before (h) and after (i) cyanobacteria growth for 10 days. Scale bars: 5 mm in (c) and 10 mm in (i).

The possibility to infiltrate and grow microorganisms in ceramic scaffolds opens the way for the implementation of bacteria‐regulated functionalities in the living material. To harness this potential, we studied the growth of cyanobacteria inside the ceramic scaffolds and evaluated the resulting living structure in terms of CO_2_‐capturing capabilities (**Figure** **4**). To quantify the growth of the microorganisms into the porous ceramic, we measured the auto‐fluorescence of the cyanobacteria in a customized experimental setup. In contrast to the fast‐growing *E. coli* (Figure 3e), the proliferation of the cyanobacteria inside the scaffold was found to initiate only after 4 days of incubation (Figure 4a). This experimental result can be explained by the doubling time of these microorganisms, which is typically 20–40 min for *E. coli* and 2.6 h for *Synechococcus sp*.^[^ ^23^ ^]^

The growth of cyanobacteria in the scaffolds is influenced by the porosity and pore size of the ceramic structure. To study this effect, we prepared porous ceramics with distinct porous architectures and measured the biomass generated in these scaffolds after bacterial growth. By combining various processing and sintering routes, three different porous architectures were prepared and investigated: 1) a low‐porosity ceramic with 23% micropores, 2) a high‐porosity scaffold with 90% macropores, and 3) a high‐porosity hierarchical scaffold with both macro‐ and micropores (93% in total). After infiltration with cyanobacteria and incubation for 13 days under artificial light, the structures were characterized in terms of biomass generated through bacterial photosynthesis (Figure 4b).

The results revealed that the presence of macropores in the two high‐porosity scaffolds increased the biomass content by a factor of 5–10 compared to the low‐porosity structure containing only micropores. Moreover, the combination of micro‐ and macropores in the hierarchical scaffold led to a two‐fold increase in biomass compared to the one‐level macroporous structure, despite the relatively similar total porosity (90–93%). While further research is required to elucidate the mechanisms underlying this effect, we expect the hierarchical porosity of the scaffold to facilitate the supply of nutrients and bacterial growth. In such a hierarchical architecture, the micropores are expected to provide the wicking function needed for liquid transport, whereas the macropores provide space for bacterial growth and serve as a reservoir of culture medium. While water evaporation from the culture medium might lead to salt precipitation within macropores during prolonged use of the scaffold, no salt accumulation was observed in our 13‐day growth experiments with cyanobacteria.

Despite the enhanced cell growth enabled by the hierarchical scaffold, the fraction of biomass synthesized by the microorganisms is significantly lower than the overall open porosity of the structure. This limited growth likely arises from the fact that cyanobacteria need light for photosynthesis, restricting biomass accumulation to the illuminated surfaces of the sample. To assess the bacterial spatial distribution across the scaffold, we cut a hierarchical sample after cell growth and inspected its cross‐section in a fluorescence microscope. The auto‐fluorescence of the chlorophyll under 625–650 nm light confirmed the preferential growth of the cyanobacteria within a 1.2–2.0 mm layer close to the surface of the scaffold. Thicker layers of biomass were found close to large crevices of the sample, which should allow for deeper penetration of light into the scaffold and thereby improve bacterial photosynthesis.

To quantify the carbon capture effect resulting from the photosynthetic process, we measured the amount of CO_2_ gas consumed by the bacteria‐laden porous structure over time (Figures 4d–f). The CO_2_ was quantified by taking aliquots of the gas phase in contact with the sample inside a customized closed chamber. In this analysis, a porous clay sample loaded with cyanobacteria was compared with a reference liquid sample containing *Synechococcus sp* at a cell density that leads to the same chlorophyll autofluorescence as in the monolith. The CO_2_ concentration in gas aliquots taken at regular intervals was measured by gas chromatography over a period of 6 h.

The CO_2_ measurements showed that the photosynthetic activity of the microorganisms enabled effective carbon capture in the bacteria‐laden scaffold. This is illustrated by the steady drop in the CO_2_ concentration in the gas phase from 600–650 to ≈250 ppm within the first 5—6 h of the experiment (Figure 4e). Carbon capture occurred 2.9‐fold faster in the cell‐laden scaffolds compared to the liquid culture of cyanobacteria (Figure 4f). Such an increase in carbon capture ability is partly related to the higher surface area of the scaffold relative to the liquid sample. Importantly, if the capturing rate is normalized over the envelope surface area, we still obtain a 1.7‐fold faster carbon capture in the monolith compared to the liquid culture. This is attributed to the higher effective surface provided by the porous microstructure of the monolith. By hosting the bacteria in a 3D structure, the scaffolds provide a means to increase the effective area of the illuminated surface while keeping a footprint comparable to that of the liquid culture medium. To evaluate the overall carbon capture performance of the bacteria‐laden structures, we compared the speed of CO_2_ uptake measured in our experiments with the capture rate previously reported for other engineered living materials. With a normalized carbon capture rate of 0.29 µmol m^−2^ s^−1^, the bacteria‐laden ceramic scaffolds display a CO_2_ consumption rate that is ≈4 times higher than that recently reported for a microalgae‐based living hydrogel.^[^ ^12d^ ^]^

The ability to 3D print structures with complex 3D shapes open the possibility to further enhance the exposure of the cyanobacteria to light and thereby the carbon capture capability of the living porous ceramic. To explore this idea, we printed a channeled structure with a twisted geometry that combines high light exposure with the high accessible surface area provided by the hierarchical porosity (Figures 4g,h). When placed on top of a bacteria‐laden culture medium, the printed structure was effectively colonized by the cyanobacteria, which formed a smooth green biofilm on the exposed surface of the monolith (Figure 4i). Interestingly, we found that the bacteria proliferate particularly well on top of the grooves created between printed filaments, which provides another geometrical parameter to increase the carbon capture capabilities of the hierarchical porous structure. It is also important to note that the porous ceramic can be easily recycled at the end of life by thermally degrading the biomass through a simple heat treatment while capturing the released CO_2_ for permanent storage.

In addition to carbon capture, the hierarchical porous ceramics provide a suitable scaffold to host bacteria for other attractive functionalities, making use of the high porosity and mechanical stability of the structures. The spectrum of possible functionalities is vast, as the tools of synthetic biology can utilize and combine diverse biological functions through genetic engineering. To illustrate the potential of this approach, we designed and tested a bacteria‐regulated living material that can detect toxic gases in the atmosphere and translate this information into a benign gas as an amplified signal that can be easily sensed by the human nose. Formaldehyde was chosen as an exemplary toxic gas that is a probable carcinogen for humans and is a well‐known indoor pollutant.^[^ ^24^ ^]^ Importantly, humans are only able to smell formaldehyde at concentrations ≈1 ppm in air,^[^ ^25^ ^]^ which is 10 times higher than concentrations that are reported to be safe against sensory irritation, lung damage, and increased cancer risk.^[^ ^26^ ^]^

**Figure 5:**
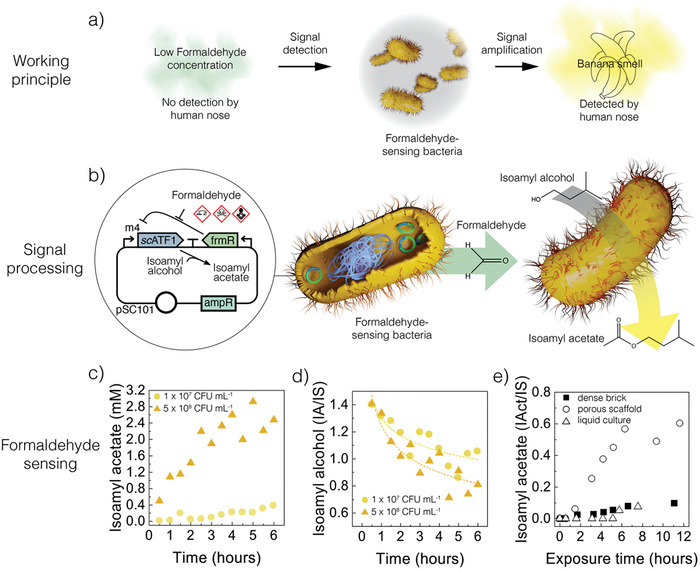
Living porous ceramic for sensing of gaseous formaldehyde. a) Working principle of the bacteria‐assisted sensing mechanism, highlighting the detection of a low concentration of toxic formaldehyde and its conversion into an amplified signal of a human‐detectable scent. b) Plasmid incorporated in the engineered bacteria to express isoamyl acetate from isoamyl alcohol triggered by the presence of formaldehyde. c,d) Concentrations of (c) isoamyl acetate generated and (d) isoamyl alcohol consumed over time in culture media containing 50 µm formaldehyde and an initial concentration of formaldehyde‐sensing E. coli of 1 × 10^7^ or 5 × 10^8^ CFU mL^−1^. e) Evolution of isoamyl acetate produced by bacteria inoculated in a hierarchical ceramic scaffold (93% porosity), in a denser sample (26% porosity), or liquid culture exposed to 1.2 ppm formaldehyde in air. The concentrations displayed in plots (d) and (e) were normalized with respect to the concentration of the internal standard (IS) 1‐propanol.

To allow humans to detect harmful formaldehyde concentrations below our olfactory capacity, we built a genetic circuit that triggers the engineered bacteria to convert isoamyl alcohol into isoamyl acetate in the presence of minor concentrations of the toxic gas (**Figure** **5**a). Isoamyl acetate is a harmless room‐temperature gas that has the scent of a banana and is easily detected by humans at concentrations as low as 0.002 mm in alcohol‐water mixtures and 0.002 ppm in the air.^[^ ^27^ ^]^ This pathway has been suggested as an attractive olfactory reporter for biosensors, due to its general absence from the environment, low toxicity, and human sensitivity.^[^ ^28^ ^]^

The genetic construct designed for formaldehyde sensing was based on a metabolic pathway used by *E. coli* for formaldehyde detoxification.^[^ ^29^ ^]^ In this pathway, the FrmR transcriptional repressor binds to its cognate promoter and thereby prevents transcription of formaldehyde‐degrading enzymes *frmA* and *frmB*. When toxic formaldehyde is present, the formaldehyde molecule binds to the FrmR protein and induces a conformational change that detaches it from the promoter, thus allowing for transcription and detoxification. We based our designs on the variant promoter m4 from plasmid pTR47m4, which was found to have higher sensitivity to formaldehyde.^[^ ^30^ ^]^ Previous work has shown that the genetic construct used for the detection of formaldehyde does not suffer from cross‐sensitivity with methanol and two other aldehydes.^[^ ^30^ ^]^ Our experiments confirmed the absence of cross‐sensitivity with methanol and also with ethanol up to 1 mm (Figure S4, Supporting Information). This high selectivity is a major advantage of the living sensor in comparison to commercially available sensors, which typically detect both ethanol and formaldehyde molecules indistinguishably. Preliminary experiments were conducted with a GFP‐expressing construct pTR47m4‐GFP, which optically confirmed the induction of the m4 promoter in engineered *E. coli* in the presence of formaldehyde gas (Figure S5, Supporting Information).

In order to express a volatile output detectable by humans, we use the enzyme ATF1 from *Saccharomyces cerevisiae*. This enzyme produces isoamyl acetate (IAct), an odorous banana‐smelling ester^[^ ^31^ ^]^ through the esterification of isoamyl alcohol (IA) with acetyl coenzyme A.^[^ ^32^ ^]^ Plasmid pFSKm4‐ATF1 was built with the ATF1 sequence placed downstream of the m4 promoter, as well as constitutively expressed FrmR, so it would express ATF1 upon formaldehyde detection.

The formaldehyde‐sensing capabilities of the engineered *E. coli* were probed by first using a liquid culture inoculated with bacteria at two different concentrations (Figures 5c,d). In this experiment, the culture medium initially contained 50 µm of formaldehyde and 10 mm of the precursor IA (Figure S6, Supporting Information). Previous experiments have shown that these formaldehyde and IA concentrations enable the production of IAct only in the presence of formaldehyde, indicating efficient repression of ATF1 in the absence of the inducer (Figure S7, Supporting Information). To assess the metabolic activity of the engineered bacteria, we measured the concentration of IAct produced and the concentration of IA consumed by the microorganisms over time.

The engineered activity of *E. coli* harboring pFSKm4‐ATF1 in a liquid medium led to a steady increase in isoamyl acetate and a decrease in isoamyl alcohol over a period of 6 h, indicating that the bacteria were able to effectively translate a weak input signal of formaldehyde (50 µm) into an amplified output signal of isoamyl acetate (up to 2.8 mm). By increasing the initial bacterial concentration by 50‐fold, from 1 × 10^7^ to 5 × 10^8^ CFU mL^−1^, we observed a 22‐fold increase in the average conversion rate within the first 3 h of the experiment (Figure S8, Supporting Information). This indicates the importance of creating scaffolds capable of hosting high bacterial concentrations to enable the detection and amplification of the formaldehyde molecule through the genetic construct.

To implement this bacteria‐assisted sensing capability in 3D structures, we colonized a hierarchical porous ceramic with the engineered *E. coli* and evaluated its ability to generate isoamyl acetate in the presence of formaldehyde in the gaseous state. In contrast to the liquid culture tests, these experiments were conducted in a customized setup to capture the conditions expected in a real application, where formaldehyde and isoamyl acetate should be detected as gases in the environment around the living scaffold (Figure S9, Supporting Information). In this experiment, the performance of the bacteria‐laden porous ceramic (93% porosity) was compared with that of a denser cell‐laden sample (26% porosity) and a liquid culture containing the same concentration of bacteria.

Porous ceramics colonized with the engineered *E. coli* were able to sense 0.12 ppm of formaldehyde gas and convert this low input signal into a human‐detectable level of isoamyl acetate (41 ppm) after 3 h of exposure to the toxic gas (Figures S9,S10, Supporting Information). Importantly, this formaldehyde concentration is 8 times lower than the odor threshold limit that can be detected by humans^[^ ^25^ ^]^ and comparable to the safe dose in terms of irritation and cancer hazards (0.1 ppm).^[^ ^26a^ ^]^ By contrast, the denser sample and the liquid culture generated significantly less isoamyl acetate when exposed to the same levels of formaldehyde (Figure 5e), which is most likely due to their lower air‐liquid interfacial area compared to the porous ceramic. The ability to host a high concentration of bacteria and distribute them over a large accessible area makes the living porous ceramic a promising platform for gas sensing of hazardous gases at room temperature with minimal energy input.

Whereas *E. coli* was used as a model organism in this demonstration, other microorganisms can also be potentially prospected and engineered to generate human‐detectable gases for chemical warning. *Actinobacteria* present in the soil are examples of microorganisms that naturally produce geosmin, known as “the smell of wet earth”.^[^ ^33^ ^]^ Since humans are sensitive to picomolar concentrations of this compound, geosmin‐producing bacteria might also be interesting microbial candidates for the fabrication of gas‐sensing living materials.

## 3 Conclusions

The colonization of porous ceramics by metabolically active microorganisms enables the creation of engineered living materials with enticing carbon‐capture and gas‐sensing functionalities. Carbon capture is achieved by hosting wild‐type photosynthetic cyanobacteria within the pores of the ceramic scaffold. This enables the fabrication of living porous structures that can remove CO_2_ directly from the air and store it as organic matter inside the cyanobacteria. For gas‐sensing, the colonizing bacteria are genetically engineered to detect toxic formaldehyde gas and transduce this chemical signal into an amplified banana scent detectably by humans. To process chemical information in a highly selective manner, the bacteria are modified with a genetic construct that is specifically designed to detect, translate, and amplify the input signal into a specific output gas. By providing room for microbial growth and transporting nutrient‐rich water, the hierarchical porosity of the ceramic scaffold is crucial for the proliferation and metabolism of such functional microorganisms. The transport of nutrient‐rich water to the microorganisms is driven by capillary forces through a spontaneous wicking mechanism that does not require external energy input for operation. Future research on hierarchical structures with graded porosity may enable further optimization of the mechanical properties, water transport, and microbial growth capabilities of the scaffold. The ability to transport water autonomously and to process chemical information in a programmable, self‐regulated manner makes bacteria‐laden porous ceramics a powerful platform for the design and creation of functional engineered living materials.

## 4 Experimental Section

### Preparation of Porous Ceramics

Porous ceramics were prepared from wet foams stabilized by clay particles.^[^ ^17b^ ^]^ The clay powder (WM‐T, Sibelco, Germany) displays an average particle size of 6 µm, a specific surface area of 15.8 m^2^ g^−1^, and a density of 2.65 g cm^−3^. According to the supplier,^[^ ^34^ ^]^ the clay consisted mostly of the mineral kaolinite mixed with inorganic impurities (Table S2, Supporting Information). The particle‐stabilized foams were generated from 35 wt% suspensions of clay (WM, Sibelco) in a water‐glycerol mixture, which was supplemented with foaming additives provided by FenX AG. The suspension was homogenized using a planetary mixer (ARE‐250, Thinky) and mechanically frothed in a 100 mL beaker for 3 min using a kitchen mixer (Multimix 5, 750 W, Braun). The previous study^[^ ^17b^ ^]^ had shown that the resulting foam displays a yield stress higher than 100 Pa and a storage modulus in the order of 10^4^ Pa, which were ideal rheological properties for extrusion‐based 3D printing.^[^ ^11^, ^35^ ^]^ Such wet foam was either cast into pillars or filled into a 50 mL syringe (BD Plastipak, BD) for 3D printing by direct ink writing (DIW).

Twisted hexagons and twisted trunk structures were 3D printed using a custom‐modified desktop printer (Ultimaker 2+). The original print head was replaced by a custom‐made extrusion system consisting of a mechanically driven syringe pump that can accommodate 50 mL syringes.^[^ ^4^ ^]^ The print paths were designed using the software Grasshopper (Rhinoceros, Robert McNeel & Associates). A nozzle with a diameter of 1.6 mm was used to print objects with a typical individual layer height of 3 mm at a print‐head velocity of 40 mm s^−1^, and an extrusion flow rate of 30 mL min^−1^.

The printed and casted parts were initially dried at room temperature for a period of 1 day, after which they were transferred to a 60 °C ventilated oven where they were dried overnight. The dried samples were then transferred to a setter plate and fired in an electrical furnace (HT 08/17, Nabertherm) using a multi‐step heating schedule. The heat treatment comprised a burnout period (200 °C for 2 h, 290 °C for 2 h, and 400 °C for 1 h) and a sintering period (1000 °C for 2 h). The heating rate was fixed at 1 °C min^−1^ for the burnout period and 4 °C min^−1^ for the sintering period. A second sintering step was employed to obtain denser porous ceramics. For this, the burnout period was skipped, and the ceramic was heated to a peak sintering temperature of 1150 °C at a heating rate of 5 °C min^−1^ and held for 1 h. The cooling rate was given by the heat loss of the oven and was not actively controlled.

### Water Uptake and Evaporation

To quantify the liquid flow within and across the porous ceramics, wicking experiments were performed on sintered pillars of ≈2.6 and 2 cm in height and diameter, respectively. The pillars were ground on their top and bottom surfaces to ensure sufficient open porosity for the wicking and evaporation of liquids. The experiments were conducted using distilled (Milli‐Q) water. During the test, the weight of the pillars was measured using the data collection software RsMulti (A&D Company, Limited, Japan) with an analytical balance (XS204, Mettler Toledo) equipped with a density kit (Mettler Toledo). For the wicking experiments, 1, 2, or 3 pillars were stacked on each other and the bottom of the stack was submerged in distilled (Milli‐Q) water up to a depth of ≈2 mm. This immersion depth was kept constant by supplying the water reservoir with additional water at a rate of 2 mL h^−1^. For the evaporation experiments, the weight of the pillars was tracked following the same procedure, starting from fully wet samples.

### Mechanical Properties

The mechanical stability of the porous ceramics at different moisture levels was evaluated by performing uniaxial compression tests on sintered pillars with a diameter and height of 2.6 and 2 cm, respectively. The compression tests were conducted on a universal testing machine (AGS‐X, 1 kN load cell, Shimadzu) at a cross‐head speed corresponding to a strain rate of 0.10 min^−1^. Before testing, the samples were ground on the top and base with sandpaper (SiC, CIMI 1000) to achieve a flat surface. The samples were pre‐conditioned before the test to be dry, partially wet, or completely wet. Dry pillars were subjected to a ventilated oven at 60 °C for a minimum of 3 h. Partially wet pillars were obtained by allowing the dry pillars to wick up deionized water for 30 min, reaching 70–80% of their total liquid imbibition capacity. Completely wet pillars were prepared by applying a vacuum to the wicked samples so that the porosity could be filled with the liquid.

### Bacterial Growth

Distinct culture media were prepared for the growth of microorganisms. A urea yeast extract medium was used to grow *Sporosarcina pasteurii*, whereas LB and HSC media were utilized to cultivate *Escherichia coli* and *Synechococcus sp*. PCC 7002, respectively. To prevent the proliferation of other undesired strains, we worked in sterile conditions. The media were filter sterilized or autoclaved, whereas the porous monoliths were thermally treated at high temperatures and were sterilized in a UV chamber before use. In the case of the cyanobacteria, the characteristic green appearance of the monoliths and liquid cultures indicate that cyanobacteria were the prominent strain. In the case of the engineered *E. coli*, the work was done with antibiotics that selected the engineered strain.

The urea yeast extract (UY) medium was prepared by adding 30 g L^−1^ urea (≥ 99.0%, ACS reagent) and 20 g L^−1^ yeast extract (YE, Millipore) in 0.13 m TRIS buffer (tris(hydroxymethyl)aminomethane, 1.0 m buffer solution at pH 9.0, Thermo Fisher). The buffer was previously diluted with deionized (Milli‐Q) water and had its pH adjusted to 7 using concentrated hydrochloric acid (37%, VWR). The media was prepared by mixing a 2x concentrated urea solution and a 2x concentrated yeast extract solution in a 1:1 ratio. The urea and yeast extract solutions had been previously sterilized by filtering and autoclaving, respectively. LB media was prepared from 20 g L^−1^ LB‐broth Lennox (Sigma–Aldrich) and 5 g L^−1^ sodium chloride (VWR chemicals) using deionized (Milli‐Q) water. High salt concentrated (HSC) media was prepared as described in the Supporting Information.

Working cultures of *Sporosarcina pasteurii* (DSM 33, Leibniz Institute DSMZ) were inoculated from a glycerol stock and grown in UY medium at 30 °C under shaking conditions (200 rpm) in an incubator (Minitron, Infors HT). Green fluorescent protein (GFP) *Escherichia coli* (DH5α bearing plasmid pKAG,^[^ ^36^ ^]^ which possesses constitutively expressed superfolder GFP) was inoculated from a single colony and grown in LB media supplemented with 50 µg mL^−1^ kanamycin at 37 °C under shaking conditions (200 rpm). Formaldehyde sensing *Escherichia coli* (DH5α bearing plasmid pFSKm4‐ATF1) was inoculated from a single colony and grown in 1 mL LB media supplemented with 100 µg mL^−1^ carbenicillin at 37 °C under shaking conditions (200 rpm). 100 µL of this pre‐culture was typically added to 10 mL media and grown overnight. *Synechococcus* sp. PCC 7002 (referred to hereafter as cyanobacteria) was cultivated in HSC media at 30 °C under 16/8 h day/night cycles and shaking conditions (150 rpm). The light intensity during the day was set to 48 µmol m^−2^ s^−1^. The cyanobacteria cultures were refreshed every 10 days by replacing 2/3 of the culture with fresh HSC media.

To assess the stage of bacterial growth in the media, the optical absorbance of the liquid cultures was measured in 96‐well plates using a microplate reader (Varioskan Lux, Thermo Scientific). The absorbance was acquired at 600 nm for *S. pasteurii* and *E. coli* and at 700 nm for the cyanobacteria. OD_600_ was calculated by pathlength correction in the Varioskan software.

### Inoculation of Ceramics with Microorganisms

Different inoculation techniques were tested to achieve high reproducibility in terms of homogeneous cell growth. The best techniques were found to be simple dipping in an overnight culture for *E. coli* or *S. pasteurii*, and dipping in a running culture in the case of the cyanobacteria. Before inoculation, the porous ceramic samples were first imbibed in fresh sterile media under vacuum to ensure that all the accessible porosity was filled with the liquid. Subsequently, the porous ceramics were inoculated by dipping them in the corresponding running culture. For incubation, the infiltrated samples were placed on top of glass beads with a diameter of ≈5 mm, which acted as spacers to allow exposure of the bottom of the sample to the fresh media.

### Bacterial Growth in Porous Ceramics

The colonization of the porous ceramics by the microorganisms was assessed following different protocols depending on the bacteria used.

The growth and viability of *E. coli* and *S. pasteurii* within the porous ceramics were quantified by estimating the concentration of viable bacteria in the samples using the most probable number (MPN) method. A pre‐treatment protocol similar to that reported by Lee et. al.^[^ ^20^ ^]^ was developed to collect bacteria from the porous ceramic structures. For this, small portions of the structures were crushed with a spatula in a tube containing a sterile solution of 0.85 wt% sodium chloride. The amount of solution was 10 times the weight of the tested sample. After vortexing at 2′700 rpm for 5 min (Vortex‐Genie 2, Scientific Industries Inc.), the tubes were sonicated in an ultrasonic bath for 6 min (130 W, Branson 1510, Branson Ultrasonics Corporation). Ice was added to the bath to prevent overheating. After sonication, the suspension was centrifuged at 1400 g for 15 min. The supernatant obtained from the centrifugation step was typically diluted to 10^−2^ to obtain sufficiently low bacteria counts and thereby reliable results with the MPN method. 20 µL aliquots of the resulting bacteria solutions were added to a 96‐well plate containing 180 µL of the adequate growth medium. Abiotic negative controls were prepared by replacing the bacteria solution with 20 µL of sterile 0.85 wt% sodium chloride solution. After serial dilutions of 1:10 up to the 8th row, the plates were incubated overnight under different conditions depending on the microorganism. After overnight incubation, positive and negative wells were scored. A well was considered positive if it became turbid after incubation. Typically, positive wells had an optical density (OD_600_) greater than 0.1. Colony‐forming units were reported as MPN mL^−1^ with 95% confidence intervals. Statistical calculations of the MPN and corresponding confidence intervals were performed using an on‐line tool developed by the United States Environmental Protection Agency (EPA).^[^ ^37^ ^]^ The input to the calculator includes the number of dilutions, the number of replicates for each sample, the amount of sample used in the most concentrated dilution, and the combination of positive and negative wells for each dilution. For the best accuracy and comparability, the number of dilutions included in the calculations must be consistent. In this work, three dilutions and a minimum of three replicates per sample were always used for the calculations.

The growth of the cyanobacteria within the porous structures was evaluated using a chlorophyll assay.^[^ ^38^ ^]^ To extract the chlorophyll, the living porous ceramic was transferred to a Falcon tube containing 1, 2, or 3 mL methanol (≥ 99.9%, Sigma–Aldrich). The resulting suspensions were shortly vortexed at 2′700 rpm and afterward incubated at room temperature for 1 h under shaking conditions at 140 rpm (Rotamax 120, Heidolph). After incubation, the porous ceramics had a pale appearance whereas the supernatant was green. Notably, structures older than 4 days required more methanol (2 or 3 mL) to extract all chlorophyll. The addition of more methanol was later considered as a dilution factor in the calculations. To quantify the amount of chlorophyll extracted from the porous ceramics, the supernatant was passed through a 0.2 µm syringe filter and the autofluorescence of the chlorophyll was measured using a plate reader in a pigmented 96‐well plate at excitation and emission wavelengths of 435 and 675 nm, respectively. The chlorophyll content of cyanobacteria cultured in media was also measured to serve as a reference. For this purpose, 100 µL of the culture was added to 1 mL of methanol, and the above procedure was repeated.

### Biocementation

Porous ceramics were mechanically reinforced through the bacteria‐induced calcification of the structure in water at room temperature. Calcification was achieved through the precipitation of calcium carbonate within the porous structure. To quantify the reinforcing effect of this biocementation process on the printed porous ceramics, abiotic and biotic twisted pentagon prints were subjected to a calcification step.

For calcification, the biotic structures were first inoculated and cultivated with *S. pasteurii* until an OD_600_ of at least 2 was reached. Following this initial cultivation step, the structures were submerged in a 40 mL calcification bath composed of 1 m calcium chloride (≥ 93.0%, Sigma–Aldrich) and 0.5 M urea (≥ 99.0%, ACS reagent) in 0.13 m TRIS buffer at pH 7. After a calcification period of 72 h at 28 °C, the structures were removed from the bath and left to dry overnight in a ventilated oven at 60 °C. Abiotic structures were prepared following the same protocol but without the initial inoculation step. Compression tests were carried out on both sets of samples in accordance with the methodology described above.

To interpret the mechanical properties of the biocemented ceramics, the amount of calcium carbonate precipitated at the end of the calcification process was also measured. The mass of precipitated calcium carbonate was quantified by performing the thermogravimetric analysis (TGA 5500, TA Instruments) of a representative portion of an abiotic and a biotic structure. The samples were ground in a mortar to a fine powder and heat treated in the air according to the following schedule: heating from 40 to 500 °C at a rate of 10 °C min^−1^, holding at 500 °C for 1 h, heating to 750 °C at a rate of 5 °C min^−1^, and holding at 750 °C for 10 min before cooling down. The mass of CaCO_3_ in the samples ($w_{CaCO_{3}}$) was determined from the mass loss between 570 and 750 °C ($\Delta w_{570-750^{\circ }C}$), which is the temperature range over which CaCO_3_ was expected to thermally decompose into CaO and CO_2_. Taking into account the stoichiometry of this reaction, the following equation was used:

(1) $$w_{CaCO_{3}}=\Delta w_{570-750^{\circ }C}\cdot \frac{M_{w}\left(CaCO_{3}\right)}{M_{w}\left(CO_{2}\right)}$$

where *M_w_* (*CaCO*_3_) and *M_w_* (*CO*_2_) are the molar mass of CaCO_3_ and CO_2_, respectively.

### Sample Imaging

Macroscopic samples were photographed using a reflex camera equipped with a macro 100 mm objective (Canon EOS 6D) at varying illumination conditions. To image the GFP *E. coli*, a UV torch was employed to illuminate the samples.

Cross‐sectional images of the porous ceramic colonized by the cyanobacteria were taken using a digital microscope (VHX‐6000, Keyence). To capture the autofluorescence of the cyanobacteria, the cross‐sections were also imaged with the ChemiDoc MP imager (Bio‐Rad) under red epi‐illumination and a 700/50 emission filter.

Electron microscopy micrographs of the porous ceramics were recorded using a field emission electron microscope (GeminiSEM 450, Zeiss) in secondary electron mode at an acceleration voltage of 3 kV, a working distance of 7.5 mm, and a probing current of 100 pA.

### CO_2_ Capture

The CO_2_ capturing capability of the cyanobacteria was quantified by measuring CO_2_ concentrations from a sealed flask over time using a gas chromatographer. For this purpose, cyanobacteria cultured for 12 days on the porous ceramic and in liquid culture were first placed inside Erlenmeyer flasks and sealed with a rubber septum. To ensure a comparable cell density in the liquid culture and in the porous ceramic, the chlorophyll content of the living porous structure was determined following the protocol described above. Based on this information, the chlorophyll content and the volume of the liquid culture were comparable to that of the porous ceramic. For both conditions, four replications were measured every 20 min. For this, the sampling volume of 4 mL was extracted from the overhead space of the flask using a 5 mL syringe. A volume of 0.5 mL was discarded, and the remaining 3.5 mL was manually injected into a gas chromatograph (Micro‐GC 3000A) equipped with a thermal conductivity detector, using He and Ar as carrier gases. To prevent the formation of an underpressure in the flask, the sampled volume was re‐injected with nitrogen gas. The dilution effect arising from the addition of nitrogen was taken into account to determine the actual CO_2_ amount captured by the bacteria.

The impact of porosity on the growth of carbon‐capturing cyanobacteria was quantified by comparing small ceramic samples with different porosities prepared from the same clay. These included a relatively dense cube subjected to a low sintering temperature (1000 °C), a porous cylinder treated at a high sintering temperature (1150 °C), and a porous cylinder subjected to a low sintering temperature (1000 °C). Such heat treatments led to porosities of 26, 90, and 93%, for the dense cube, the porous cylinder sintered at 1150 °C and the porous cylinder sintered at 1000 °C, respectively. To enable bacteria colonization, the samples were inoculated and incubated for 13 days. The biomass of the resulting living structures was quantified from the loss of ignition (LOI) of the dried samples. The LOI corresponds to the amount of organic mass lost by the sample during thermal treatment. To measure the LOI, the samples were heated to 550 °C at a rate of 4 °C min^−1^ and held at this peak temperature for 5 h. A high‐precision scale (0.01 mg readability, XP205, Mettler Toledo) was employed to determine the weight loss of the heat‐treated samples.

### Plasmid Design and Construction

Plasmid pFSKm4‐ATF1 was based on the backbone of pTR47m4‐GFP (Addgene #102436),^[^ ^30^ ^]^ containing the pSC101 origin of replication, ampicillin resistance and the repressor *frmR* under the regulation of the *ptet* promoter, which was constitutive since the host *E. coli* DH5a lacked a *tetR* repressor. The ATF1 sequence was obtained from plasmid p006‐Banana‐late (Addgene #112251),^[^ ^39^ ^]^ and included the moderate ribosome binding site BBa_R0064. This ATF1 sequence was used to replace the RBS and coding sequence of sfGFP on plasmid pTR47m4‐GFP to create plasmid pFSKm4‐ATF1. Physical DNA and full sequence information of pFSKm4‐ATF1 could be found at Addgene (Plasmid #221133).

DNA construction was performed using Gibson assembly,^[^ ^40^ ^]^ following the protocol in reference using enzymes Taq ligase (M0208S), T5 Exonuclease (M0663S), and Phusion polymerase (M0530S), obtained from New England Biolabs (NEB). PCR fragments were obtained using custom DNA oligos (Integrated DNA Technologies) on the templates specified, designed to contain 25–30 bp overlaps between assembled fragments. PCR was performed using the Q5 Hot Start High‐Fidelity (M0494S) from NEB. PCR fragments were purified with Reliaprep DNA Clean‐Up and concentration system (A2892, Promega). Cloning was performed in E. coli DH5a chemically competent cells. Plasmid purification was performed with the PureYield Plasmid Miniprep System (A1223, Promega). Plasmid DNA sequence was confirmed by Sanger sequencing carried out by MicroSynth, Switzerland.

### Formaldehyde Sensing

The ability of the engineered *E. coli* to sense formaldehyde was evaluated by means of headspace gas chromatography–mass spectrometry (GC‐MS) (Trace 1300 and ISQ, Thermo Scientific). Because the formaldehyde sensing capability was expected to depend on bacterial density, experiments were first prepared with liquid cultures inoculated with controlled initial concentrations of engineered *E. coli*. Inoculum cultures were prepared by picking and growing colonies of *E. coli* bearing pFSKm4‐ATF1 overnight in LB media with 100 µg mL^−1^ carbenicillin at 37 °C under shaking conditions. This yielded turbid cultures with an OD_600_ of 2.1, which corresponded to 1.1×10^9^ CFU mL^−1^. Cells from overnight cultures were harvested by centrifugation and redispersed at varying proportions in fresh LB media supplemented with 100 µg mL^−1^ carbenicillin and 10 mm isoamyl alcohol (> 99.0%, Fluka). This concentration of isoamyl alcohol was found to be a good compromise between not inhibiting bacterial growth and providing enough substrate for ATF1 (Figure S11, Supporting Information). In the presence of formaldehyde, the engineered *E. coli* converted isoamyl alcohol into isoamyl acetate.

The impact of bacterial density on the resulting isoamyl acetate concentration was evaluated by the addition of 50 µm formaldehyde to freshly redispersed cultures inoculated with either 1 × 10^7^ or 5 × 10^8^ CFU mL^−1^ respectively. During incubation of the cultures at 30 °C and 200 rpm, small aliquots (200 µL) were sampled at given time intervals and placed in 10 mL headspace vials. 10 µL 1‐propanol (99+%, Acros organics) was added to the vials before sealing to quench the bacteria activity and to serve as an internal standard for the quantification with GC‐MS. The vials were thoroughly mixed, sonicated for 1 min, and left to rest for 15 min before 0.5 mL of the headspace was sampled and injected into the gas chromatographer. To quantify the isoamyl acetate concentration, the chromatogram was evaluated at selected masses at which isoamyl acetate, isoamyl alcohol, and 1‐propanol peaks are expected (*m/z* = 30 and *m/z* = 70). The ratio of the isoamyl alcohol and isoamyl acetate peak integrals to the 1‐propanol peak integral (internal standard) was calculated and used for quantification. The isoamyl acetate concentrations were obtained from a calibration curve (Figure S6, Supporting Information).

To evaluate the formaldehyde‐sensing ability of the engineered *E. coli* inside living ceramics, experiments were designed with dense or porous samples using a liquid culture as a control. The dense and porous samples featured porosities of, respectively, 26 and 93%, and were prepared in the same as those used for the growth of cyanobacteria. To colonize the porous and dense structures with *E. coli*, samples were dipped into LB media (with 10 µg mL^−1^ carbenicillin and 10 mm isoamyl alcohol) inoculated with 1 × 10^9^ CFU mL^−1^ and infiltrated by applying a vacuum for 20 s. The volume of the liquid culture used as a control was the same as the one wicked by the porous ceramic. For the quantification of the sensing ability of the living ceramics, the samples were exposed to formaldehyde through the gas phase, and gas aliquots were extracted for GC‐MS measurements. 3 mL of aqueous solutions with 1.2 and 12 mm formaldehyde and 50 or 150 µL 1‐propanol as internal standard was added to an Erlenmeyer flask into which a smaller vial containing the sample was placed. Using Henry's law, the equilibrium concentrations of formaldehyde in the gas phase around these solutions were estimated to be 125 and 1250 ppb. All samples were sealed with a rubber septum and left still at room temperature during the collection of gas aliquots. At given time intervals, an aliquot of 0.5 mL was then taken from the headspace of the Erlenmeyer flask and injected into the GC‐MS. In the absence of a calibration curve for all samples, the isoamyl acetate concentrations were reported relative to the internal standard (Figure S9, Supporting Information).

## Conflict of Interest

The authors declare no conflict of interest.

## Author Contributions

The concept of this study was developed by Alessandro Dutto, Anton Kan, and André R. Studart. The porous ceramic monoliths and prints were prepared by Alessandro Dutto, Zoubeir Saraw, and Aline Maillard. Zoubeir Saraw conducted the water uptake, bacterial proliferation, and mechanical studies. Alessandro Dutto and Aline Maillard investigated the growth and carbon‐capturing capability of the cyanobacteria. Alessandro Dutto and Daniel Zindel developed the methods for the gas sensing setup. Anton Kan engineered the gas‐sensing E. coli and performed the tests for toxicity and cross‐sensitivity. The figures were prepared by Alessandro Dutto and revised by André R. Studart. The text was written by André R. Studart and Alessandro Dutto and was revised by all authors.

## Supporting information



Supporting Information

## Data Availability

The data that support the findings of this study are available from the corresponding author upon reasonable request.
