# Supplementary material for: Living Porous Ceramics for Bacteria‐Regulated Gas Sensing and Carbon Capture
Source: Adv Mater. 2024 Dec 10;37(5):2412555. doi: 10.1002/adma.202412555 (PMC11795706; doi:10.1002/adma.202412555)
Supplement: Supplementary file 1 — Supporting Information [file ADMA-37-2412555-s001.docx]

**Supplementary information**

**Living porous ceramics for bacteria-regulated gas sensing and carbon capture**

Alessandro Dutto, ^1^ Anton Kan, ^1^ Zoubeir Saraw, ^1^ Aline Maillard, ^1^ Daniel Zindel, ^2^ André R. Studart ^1^

^1^ Complex Materials, Department of Materials, ETH Zürich, 8093 Zürich, Switzerland

^2^ Laboratory of Physical Chemistry, ETH Zürich, 8093 Zürich, Switzerland

**Supplementary text**

**Estimation of water transport in porous structures**

In analogy to the water transport in trees, the transport of liquid through the pores of a partially wetted porous object is driven by the evaporation at the surface of the object and the cohesion forces of water. Molecular cohesion enables the transport of water over long distances within porous structures through the action of capillary forces. Such forces allow for the vertical transport of water even against the action of gravity. Surface evaporation from a liquid-saturated porous object is often the rate-limiting step that controls the speed of liquid transport through the structure. Here, we estimate the maximum vertical distance that water would be able to travel in our porous ceramics and the expected travelled distance in these structures in a possible application.

To estimate the maximum vertical distance travelled by water inside the porous ceramics, we use the idealized model of a vertical tube partially filled with a wetting liquid. Assuming a tube of radius $r_{0}$, the vertical distance ($h$) travelled by water in the tube through capillary action can be estimated by applying Jurin’s law: ^[1]^

$h=\frac{2\gamma\cos\theta}{\rho gr_{0}}$ (1)

where $\gamma$ is the surface tension of the wetting liquid, $\theta$ is the contact angle of the liquid on the solid wall of the tube, $\rho$ is the density of the wetting liquid and $g$ is the gravitational acceleration constant ($g=9.81\frac{m}{s^{2}}$). Taking water as the wetting liquid and considering the hydrophilic nature of the clay, we assume $\gamma=72\frac{mN}{m}$, $\theta=10^{\circ}$, and $\rho=1000\frac{kg}{m^{3}}$. The radius, $r_{0}$, can be approximated by the size of the micropores of the porous ceramic, which typically ranges from $20$to $80 nm$. ^[2]^ Provided that these pores are interconnected, Jurin’s equation results in theoretical vertical distances ranging from $180 m$ to $720 m$. This estimation clearly shows that the transport of liquid in such porous structures is dominated by surface forces rather than gravity.

While capillary forces enable water transport over very large vertical distances, it is important to note that the timescale for transport is also strongly dependent on the characteristic size of the pores. Such dependence of the wicking timescale on pore size is described by Washburn’s equation:

$t=h^{2}\frac{2\eta}{r_{0}\gamma\cos\theta}$ (2)

where $\eta$ is the viscosity of the liquid. For water, $\eta=0.89 mPa\cdot s$. From this equation, one can predict that for pore sizes of 20-80 nm it would take 1 year for liquid to be transported along 5-10 m. This estimation indicates that the infiltration of the porous structure through immersion in liquid is the most time-effective approach to saturate the pores with the liquid phase. After this initial infiltration step, the water-saturated porous structure should enable autonomous liquid transport driven by capillary forces.

To keep the porous structure saturated with nutrient-rich liquid, the wicking rate needs to be comparable or higher than the evaporation rate. Our experiments on centimeter-scaled porous monoliths resulted in specific evaporation rates ranging from $97$ to $125\frac{g}{h m^{2}}$ and specific wicking rates ranging from $32$ to $84\frac{kg}{h m^{2}}$. These data provide useful guidelines for the design of brick elements, since it allows us to calculate the geometry that would ensure that the surface of the porous monolith remains wet (saturated condition). Based on our experimental data, the wicking rate will be higher than the evaporation rate if the ratio between exposed surface and cross-sectional area of the wall is equal or lower than approximately 500. For a brick element with a wicking cross-sectional area of 20x20cm^2^, it should be possible to keep the porous structure saturated with liquid if the evaporation surface is kept below 20m^2^.

Since these are reasonable dimensions for architectural walls and facades, our analysis suggests that our porous ceramics provide a suitable scaffold for the transport of nutrient-rich water for the growth of microorganisms in building elements.

**High salt concentrated (HSC) media**

The HSC media used for the cultivation of *Synechococcus sp* was prepared by mixing ASNIII and BG11 media in a 1 to 1 volume ratio.

The ASNIII medium was prepared using a standard recipe provided by the supplier (ATCC medium: ASN-III medium). 80% of deionized (MilliQ) water was first added to a beaker and heated to 50°C under stirring. The chemicals in Table S3 were added, the heater was turned off, and the solution left to stir over night at 600 rpm. Cyanocobalamin (Acros organics) was then added from a stock solution to reach a concentration of 10 µg mL^-1^ and the remaining water was topped up. The media was autoclaved and let cool down below 60°C before the trace metal mix (Table S4) was added in a trace metal mix: media volume ratio of 1:1000.

The BG11 medium was prepared by diluting the BG11 100x solution purchased from Merck with sterile deionized (MilliQ) water at a volume ratio of 1:100. The resulting solution was supplemented with 1:1000 trace metal mix (Table S4).

**Supplementary figures**


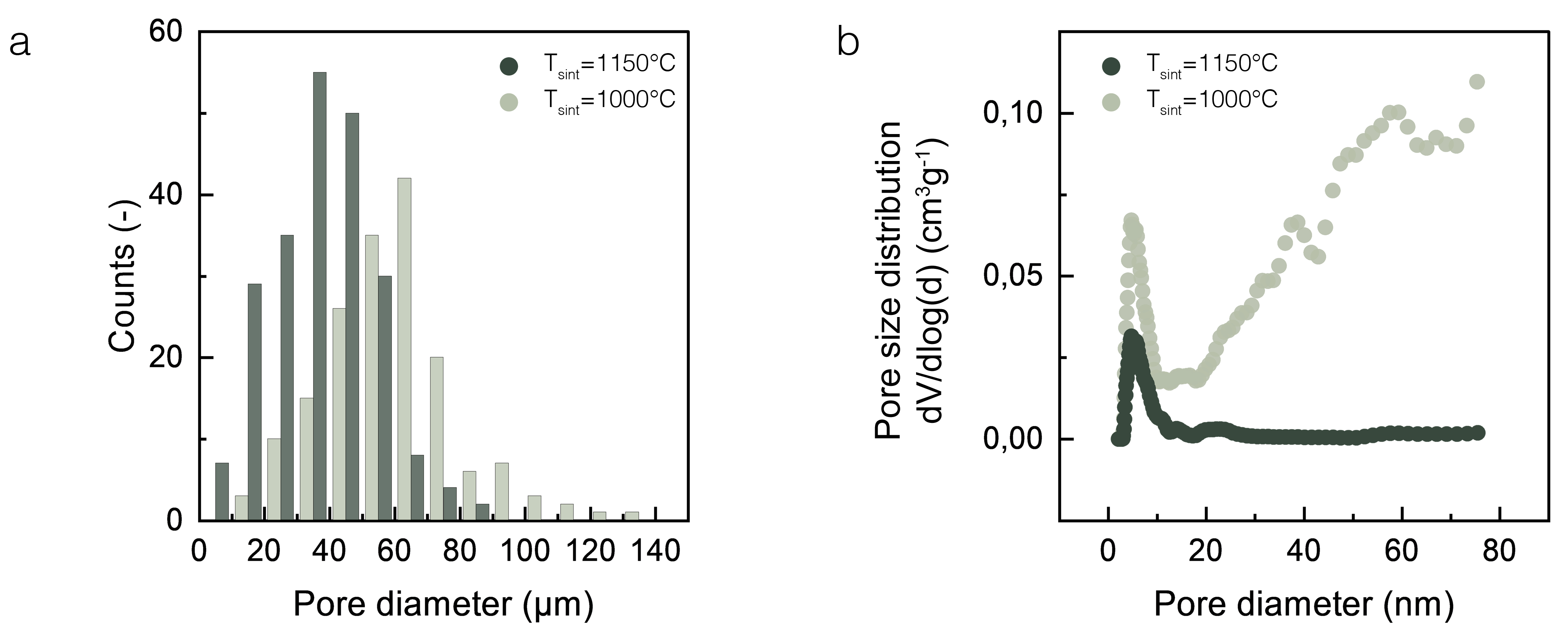


**Figure S1.** Size distribution of (a) macro- and (b) micropores of ceramic scaffolds sintered at distinct temperatures. The macroporosity data were measured by image analysis of microscopy images, whereas the size distribution of micropores were obtained from previously reported data. ^[2]^

**
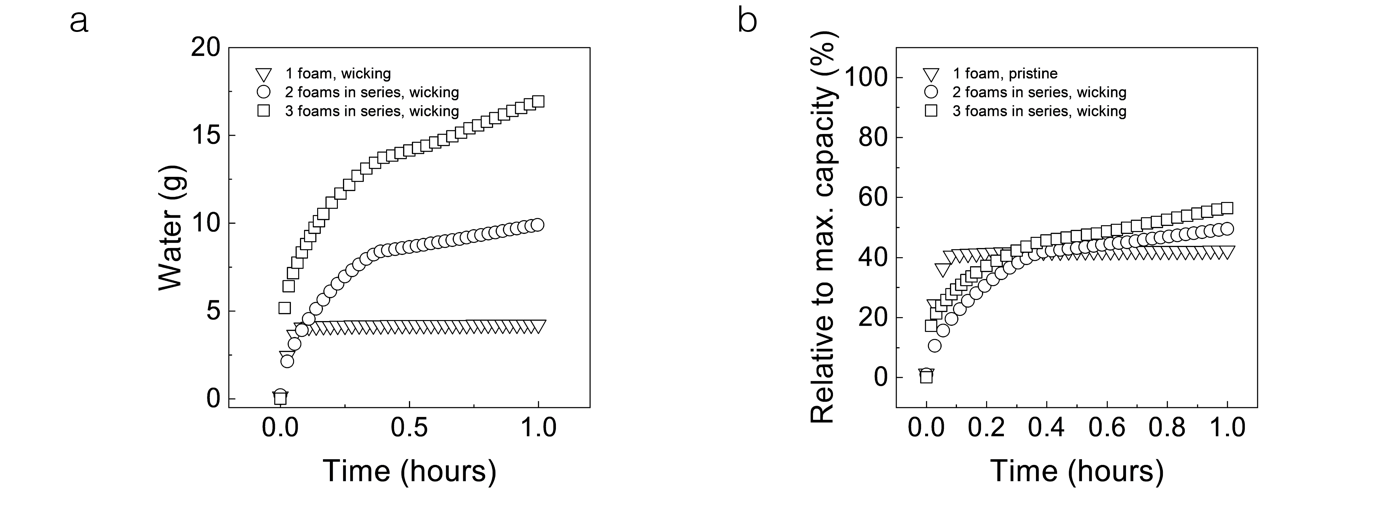
**

**Figure S2.** **Wicking behavior of 1, 2 and 3 porous monoliths in series.** Water uptake in absolute values (a) and relative to the maximum capacity of the pillar system (b). The maximum capacity was determined by infiltrating the structures under vacuum. By stacking multiple porous ceramic pillars on top of each other, more relative porosity can be filled up within the first hour. The meniscus forming at the interface between two pillars grows at a slower rate given its larger curvature. This additional wicking force is reflected in the larger slope for times longer than 0.4 hours in the case of the stacked systems, which is 0.05, 2.5, 5.5 mL/h in the case of 1, 2 and 3 pillars, respectively.

**
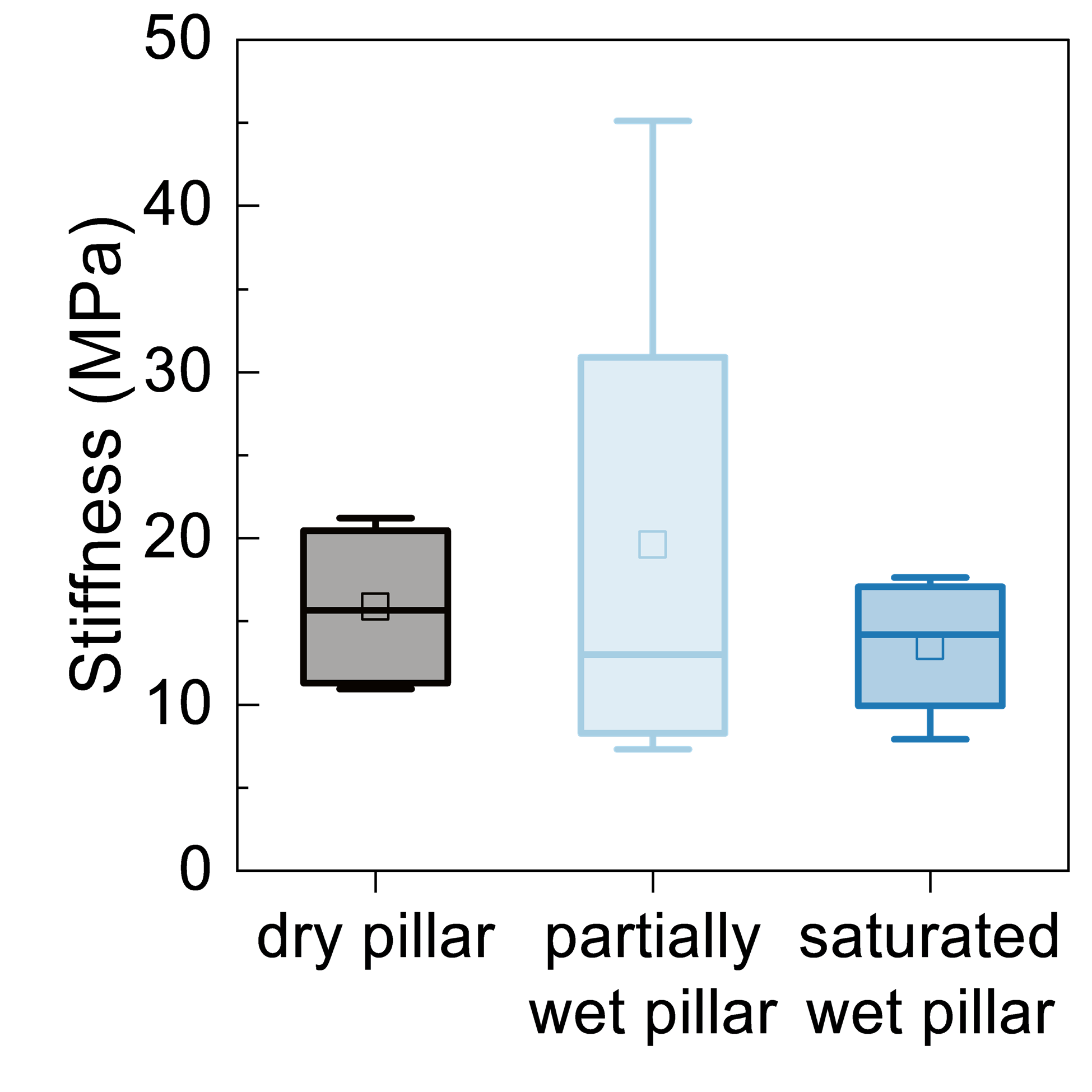
**

**Figure S3.** **Stiffness of cylindrical scaffolds in the dry, partially wet and fully wet states.**

**Figure S4. Formaldehyde sensor cross-reactivity.** *E. coli* DH5a bearing plasmid pTR47m4-GFP were initially grown overnight in LB media with 100 µg mL^-1^ carbenicillin at 37°C in a shaking incubator, and this starter culture was used to inoculate LB media containing varying concentrations of ethanol, methanol, or formaldehyde. Cultures were grown in 96-well plates at 30°C in shaking conditions. No GFP activation was observed with ethanol or methanol. No cell growth was observed at the highest formaldehyde concentration tested.

**
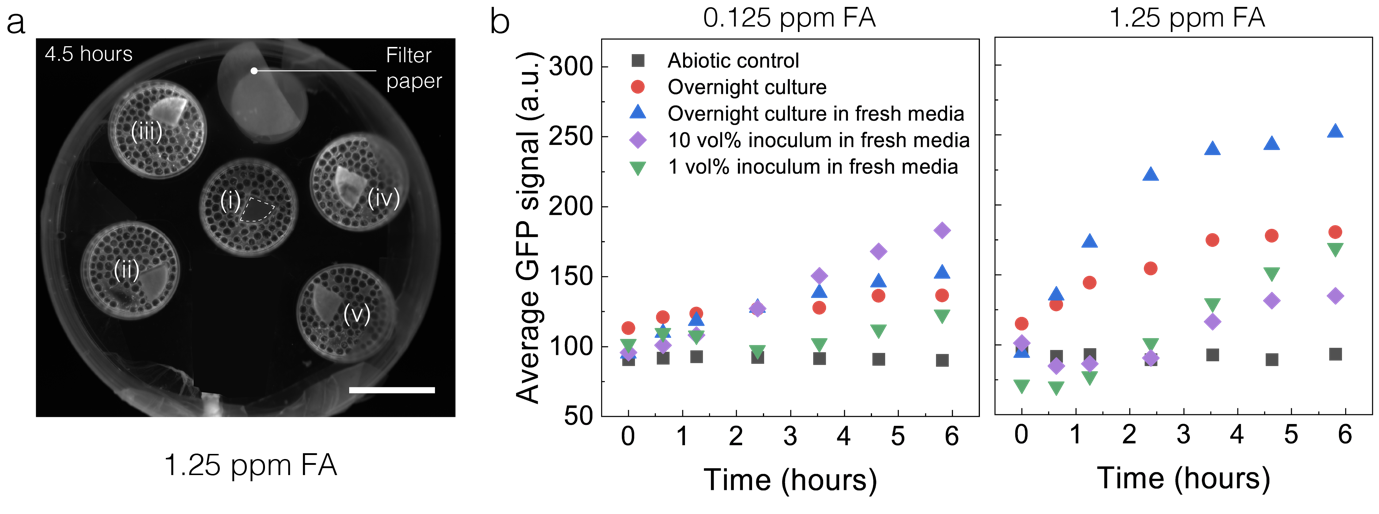
**

**Figure S5.** **Bacterial sensing of formaldehyde in the gas phase.** (a) Selected fluorescence image showing the experimental setup and the GFP signal of an abiotic control (i) and living porous ceramics infiltrated with an overnight culture (ii), an overnight culture redispersed in fresh medium (iii), 10 vol% inoculum in fresh media (iv) and 1 vol% inoculum in fresh media (v) of engineered *E. coli* exposed to 1.25 ppm of formaldehyde for 4.5 hours. Scale bar 5 cm. (b) GFP signal for living ceramics populated by different amounts of engineered *E. coli* exposed to 0.125 ppm (left) and 1.25 ppm (right) formaldehyde*.* The concentration of formaldehyde was estimated using Henry’s law (see Figure S6).


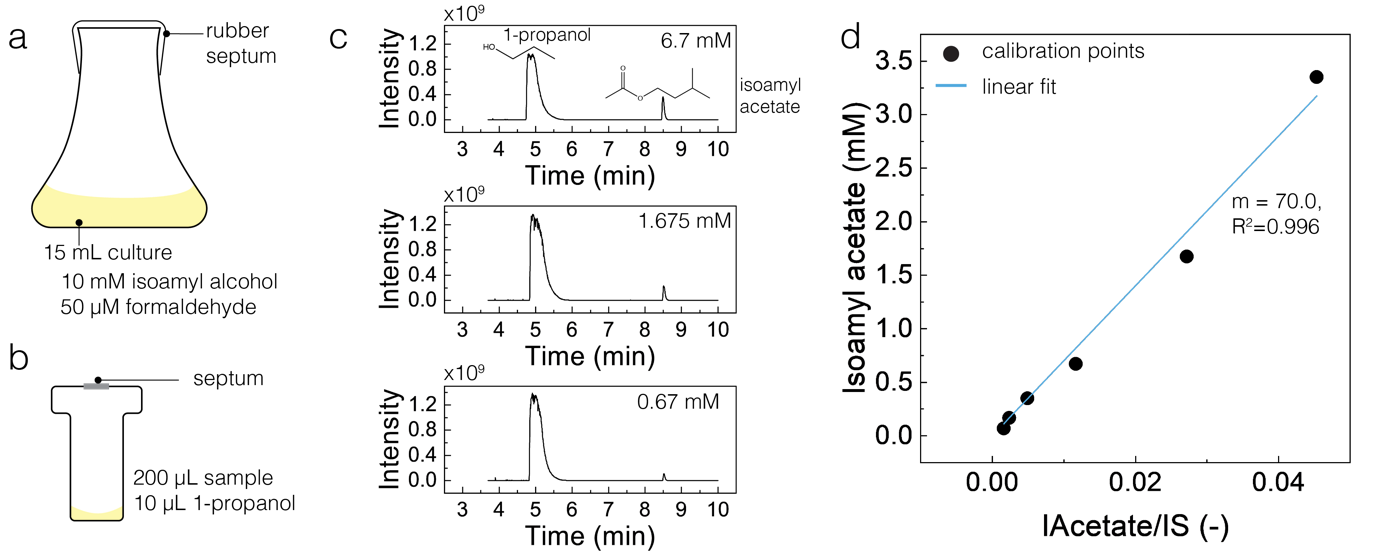


**Figure S6. Bacterial sensing of formaldehyde in the liquid phase.** (a) Schematic of the Erlenmeyer used to incubate the engineered *E. coli* DH5a bearing plasmid pFSKm4-ATF1 in the presence of formaldehyde for the conversion of isoamyl alcohol into isoamyl acetate. (b) Schematic of the headspace vial to measure the concentration of isoamyl acetate. (c) Selection of chromatographs for isoamyl acetate concentrations of 6.7, 1.675 and 0.67 mM. The first peak (RT = 4.9 min) is the internal standard (IS) 1-propanol and the smaller one (RT = 8.5 min) is isoamyl acetate (IAcetate). The retention time of each molecule was confirmed by the mass spectra. (d) The calibration curve was obtained by measuring the ratio of the peak integrals of IAcetate and IS for solutions containing known concentrations of isoamyl acetate in (c). The linear fit gives a slope (*m*) of 70.0 mM (R-square = 0.996).


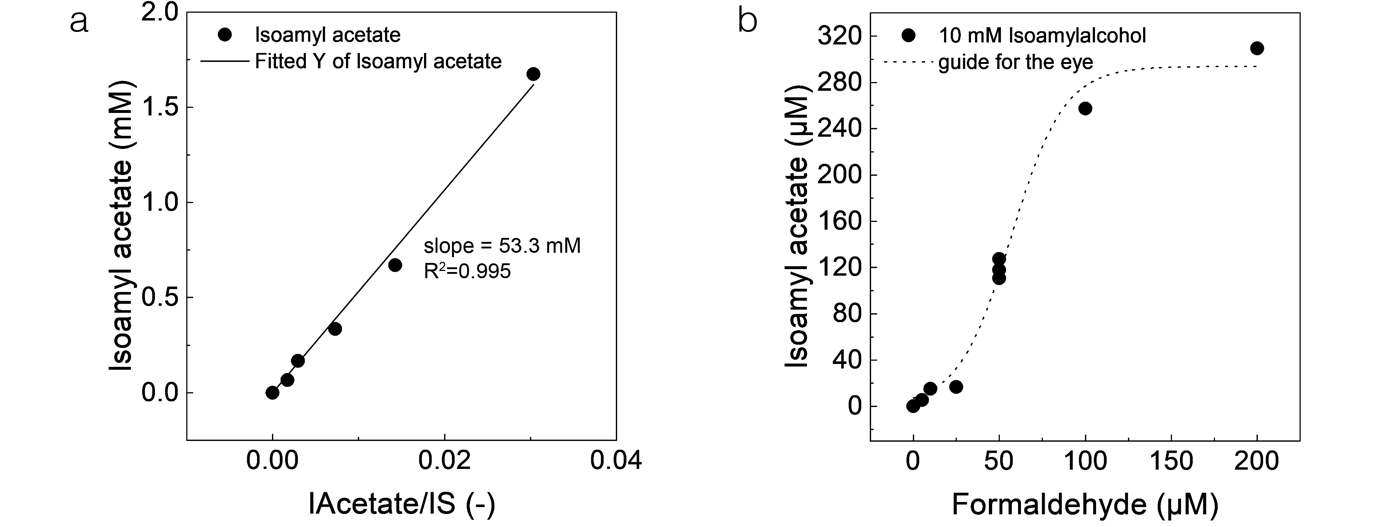


**Figure S7.** **Bacterial growth and sensitivity with formaldehyde in liquid.** *E. coli* DH5a bearing plasmid pFSKm4-ATF1 was initially grown overnight in LB media with 100 µg mL^-1^ carbenicillin at 37°C in a shaking incubator, and this starter culture was used to inoculate LB media containing 10 mM isoamyl alcohol and varying concentrations of formaldehyde. Cultures were grown at 30°C in shaking conditions. (a) Calibration curve used to estimate the isoamyl acetate concentration. The internal standard 1-propanol was used. (b) Isoamyl acetate concentrations after 2 days growth in contact with formaldehyde. No isoamyl acetate activation was observed in absence of formaldehyde.


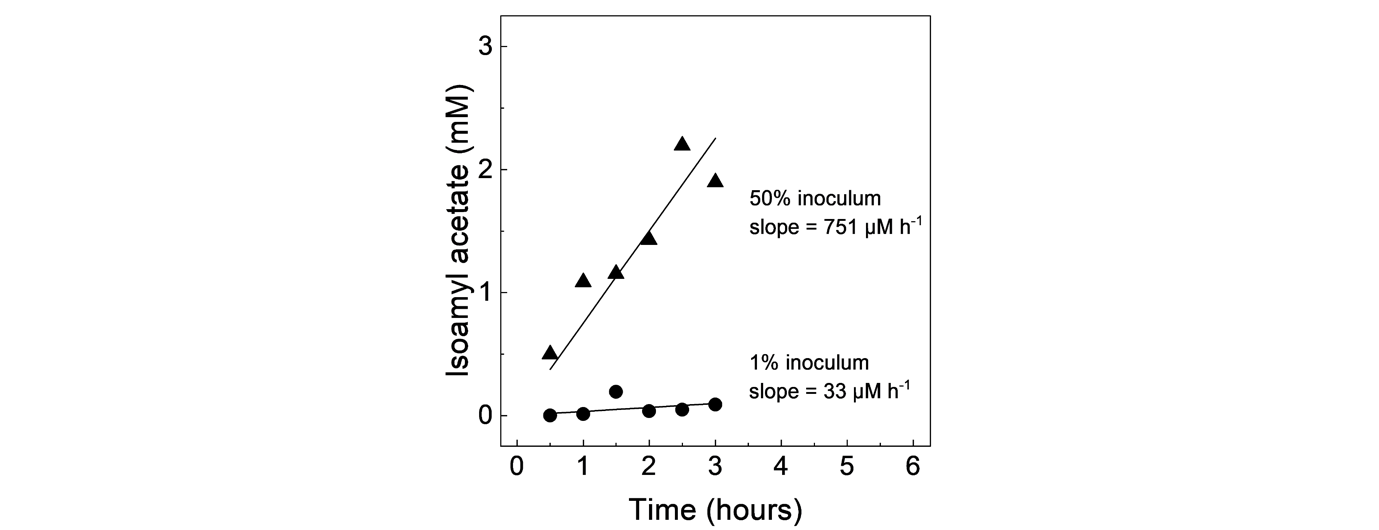


**Figure S8.** **Bacterial sensing of formaldehyde in liquid.** Conversion rates for 1% and 50% inoculum in the first 3 hours of the experiment shown in Figure 5c.


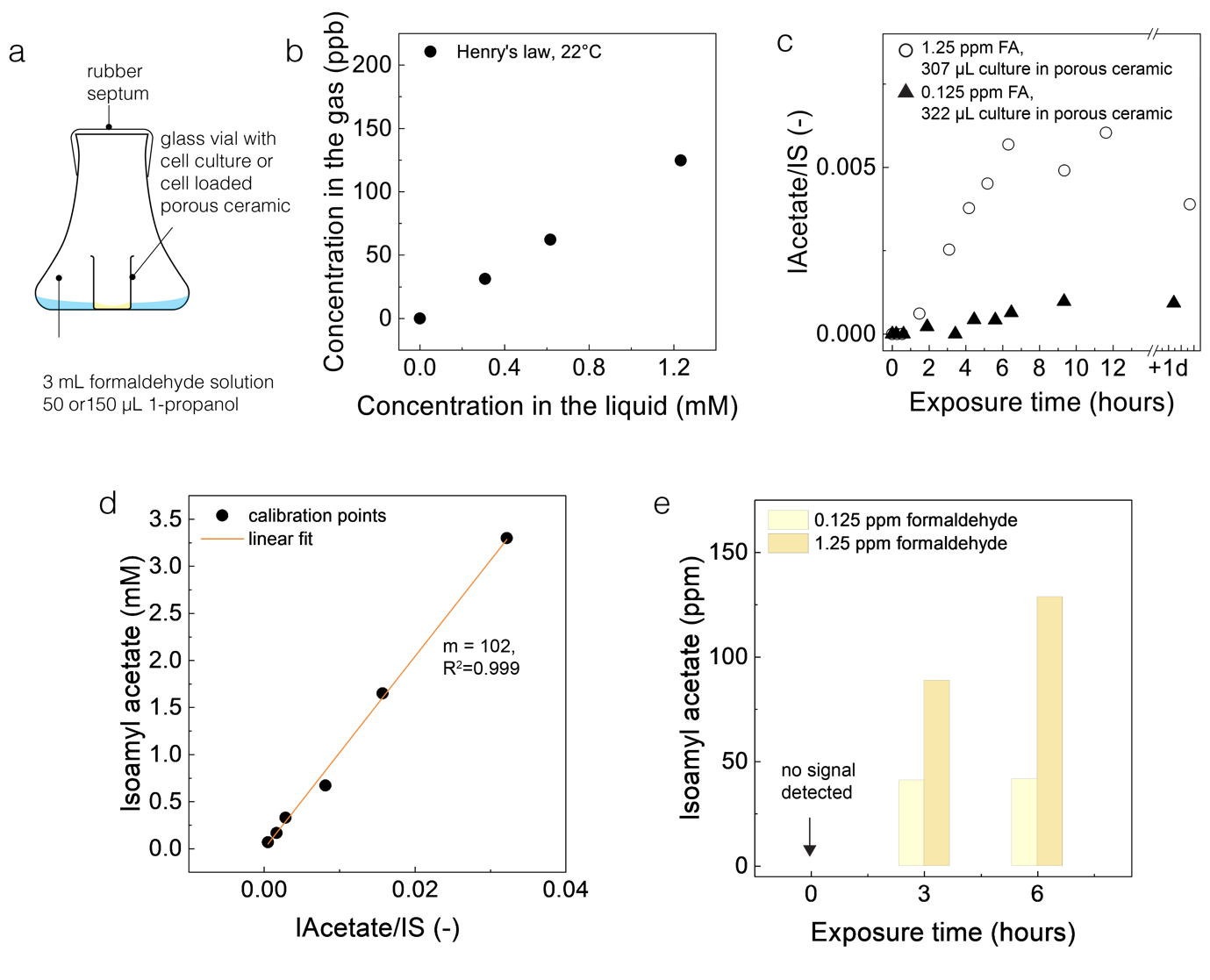


**Figure S9.** **Bacterial sensing of formaldehyde in the gas phase.** (a) Schematic of the Erlenmeyer from which headspace samples were taken. (b) Estimation of the concentrations of formaldehyde in the gas phase based on the partial pressure obtained from Henry’s law: $p=H\cdot c$, where $p$ is the partial pressure of formaldehyde, $c$ is the concentration of formaldehyde in the liquid and the proportionality constant $H_{FA}= 0.1{atm cm}^{3}\mathrm{mol}^{-1}$ is taken from values reported in literature for formaldehyde in water at 22°C. ^[3]^(c) Isoamyl acetate (IAct) concentration relative to the internal standard (IS, 150 µL 1-propanol) for two porous ceramics containing the engineered *E. coli* DH5a bearing plasmid pFSKm4-ATF1 exposed to 125 and 1250 ppb formaldehyde (1.2 and 12 mM in the solution). (d) Calibration curve for the setup shown in (a) using 50 µL 1-propanol as internal standard. The linear fit gives a slope (*m*) of 102 mM (R-square = 0.999). (e) Isoamyl acetate concentration in the air after 0, 3 and 6 hours exposure time. The concentration in the air was estimated using Henry’s law with the proportionality constant $H_{IAct}= 113.6{atm cm}^{3}\mathrm{mol}^{-1}$ taken from literature values. ^[4]^


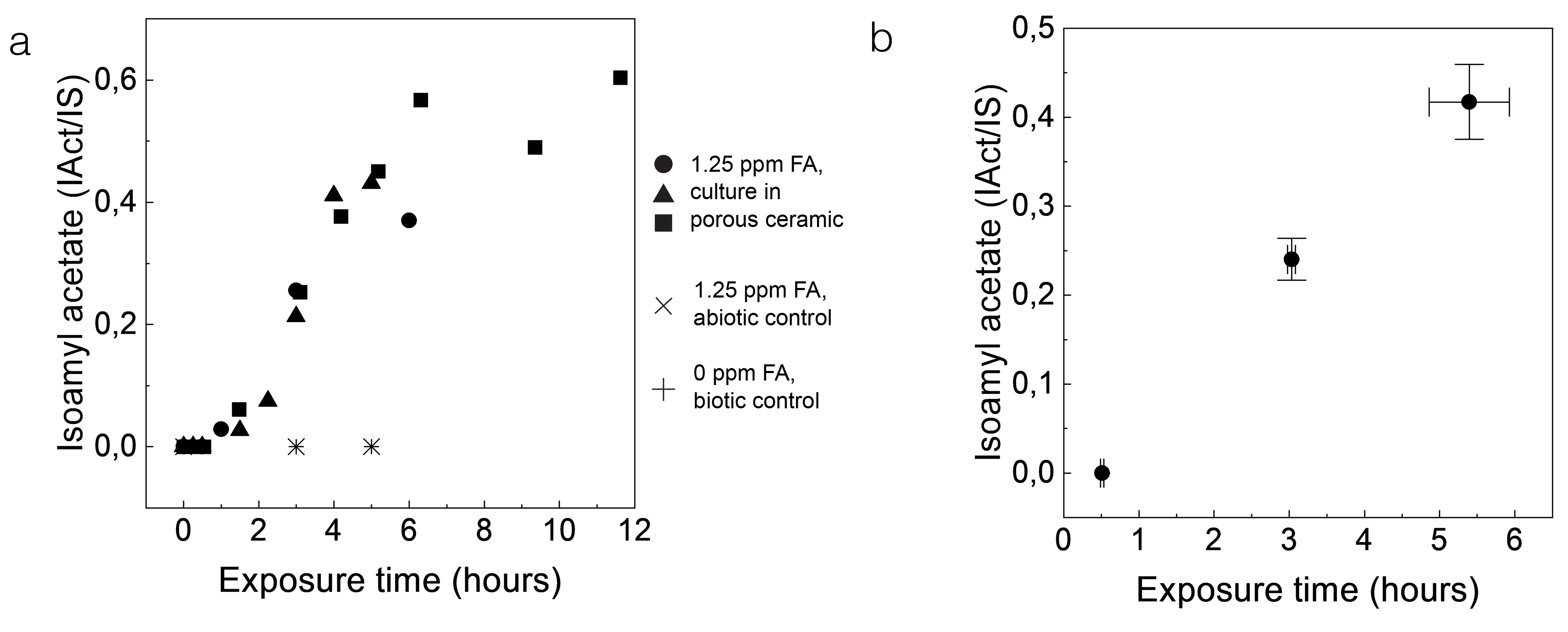


**Figure S10.** **Reproducibility of bacterial sensing of formaldehyde in the gas phase.** (a) Time evolution of the isoamyl acetate (IAct) concentration relative to the internal standard (IS) of three distinct porous ceramics under the following conditions: (i) sample containing the engineered *E. coli* DH5a bearing plasmid pFSKm4-ATF1 exposed to 1.25 ppm formaldehyde (FA), (ii) abiotic control samples exposed to 1.25 ppm FA, and (iii) biotic negative control sample without FA. (b) Evolution of the average relative isoamyl acetate concentrations (IAct/IS) obtained from the three independent samples containing the engineered E. coli DH5a bearing plasmid pFSKm4-ATF1 exposed to 1.25 ppm FA. The error bars indicate the standard deviation.

**Figure S11. Isoamyl alcohol toxicity for E. coli DH5a bearing plasmid pFSKm4-ATF1.** Bacteria were initially grown overnight in LB media with 100 µg mL^-1^ carbenicillin at 37°C in a shaking incubator, and this starter culture was used to inoculate LB media containing a variable concentration of isoamyl alcohol, which was grown at 30°C under shaking conditions. No cell growth was detected above 30 mM.

**Supplementary tables**

**Table S1.** Evaporation rates of the porous monoliths calculated from the water loss data (Figure 3b).

| Monolith | Evaporation rate (g/h) | Exposed surface area (m^2^) | Specific evaporation rate  (g.h^-1^.m^-2^) |
| --- | --- | --- | --- |
| 1 unit | 0.32 | 0.00273 | 117.2 |
| 2 units | 0.41 | 0.00422 | 97.1 |
| 3 units | 0.76 | 0.00608 | 125.0 |

**Table S2.** Chemical composition of the clay powder (WM-T, Sibelco).

| Chemical analysis (%) | SiO_2_ | TiO_2_ | Al_2_O_3_ | Fe_2_O_3_ | CaO | MgO | K_2_O | Na_2_O |
| --- | --- | --- | --- | --- | --- | --- | --- | --- |
|  | 75.2 | 1.50 | 19.4 | 0.80 | 0.20 | 0.40 | 2.30 | 0.20 |
| Loss of ignition (%) | 5.8 | | | | | | | |
| Moisture (%) | 20.0 | | | | | | | |

Data provided by the supplier (https://shop.sibelco.com/global/en/EUR/p/600002649/PB%20WM-T%20PO)

**Table S3.** Ingredients for the ACSNIII media.

| **Concentration (g L^-1^)** | **Ingredient** |
| --- | --- |
| 25 | Sodium chloride (VWR chemicals) |
| 0.95 | Magnesium chloride (Abcr) |
| 0.5 | Potassium chloride (VWR chemicals) |
| 0.02 | Di-potassium hydrogen phosphate trihydrate (p.a., Merck) |
| 3.5 | Magnesium sulfate heptahydrate (≥99.0%, Sigma Aldrich) |
| 0.5 | Calcium chloride dihydrate (VWR chemicals) |
| 0.003 | Citric acid (Sigma Aldrich) |
| 0.0005 | Ethylenediaminetetraacetic acid (EDTA) (≥99%, Roth AG) |
| 0.04 | Sodium carbonate (Merck) |
|  | Deionized (MilliQ) water |

**Table S4.** Ingredients of the trace metal mix.

| **Concentration (g L^-1^)** | **Ingredient** |
| --- | --- |
| 2.86 | Boric acid (≥99.5%, Sigma Aldrich) |
| 1.81 | Manganese(II) chloride tetrahydrate (99+%, Acros Organics) |
| 0.222 | Zinc sulfate heptahydrate (≥99.5%, Sigma Aldrich) |
| 0.390 | Sodium molybdate(VI) dihydrate (99+%, Acros Organics) |
| 0.079 | Cupric sulfate pentahydrate (≥99.0%, Fluka Chemie AG) |
| 0.049 | Cobalt(II) nitrate hexahydrate (≥98%, Sigma Aldrich) |
|  | Deionized (MilliQ) water |

**References**

[1] a)J. Jurin, *Philosophical Transactions of the Royal Society of London* **1718**, 30, 739; b)S. Liu, S. Li, J. Liu, *The European Physical Journal E* **2018**, 41, 46.

[2] A. Dutto, M. Zanini, E. Jeoffroy, E. Tervoort, S. A. Mhatre, Z. B. Seibold, M. Bechthold, A. R. Studart, *Advanced Materials Technologies* **2022**, 10.

[3] B. R. Kim, E. M. Kalis, T. DeWulf, K. M. Andrews, *Water Environment Research* **2000**, 72, 65.

[4] R. Sander, *Atmospheric Chemistry and Physics* **2023**, 23, 10901.
